# Surgery

**Published:** 1876-05

**Authors:** 


### Summary of Progress in the Medical Sciences.

### I. Surgery.

1. *Fatty Embolism as a Cause of Sudden Death after Injuries of the Bones.* (*The Med. Record*, New York, Jan. 29, 1876 ; *Berlin Klin. Wochenschrift*, Nov. 1 and 8, 1875.)

Prof. V. Czerny, of Freiburg, taking the reports of two cases as a text, discusses this subject mainly in its clinical aspects. The first case was that of a healthy man, aged 24, who, Nov. 16, sustained a simple fracture of the right thigh in its upper third, caused by falling off a sand bank. The surgeon who was called to him, found that he suffered little pain at the seat of the fracture, but could not lie down without difficulty, especially on his back, and could not breathe easily.

On the 18th, the swelling having subsided, a pasteboard splint was applied, the patient at the time finding it very difficult to maintain a half-sitting posture. On the night of the 20th, great dyspnœa and depression suddenly manifested themselves, without inflammation, and he died on the 22nd.

The authorities subsequently had the body disinterred and examined. The fragments of bone were in proper position, but both lungs were full of venous blood, and the membranous substance, and blood vessels of the brain also, were similarly filled. He cites the opinion of Wagner, who, in 1865, states that a sudden fatty embolism was capable of inducing hyperæmia of the lungs, œdema and death ; and in 1866, of Busch, who established that the medulla of the bones, when crushed, was taken up by the veins and lymphatics, and caused emboli in the lungs, and death.

Czerny's second case is as follows :

J. S., aged 32, a strong and healthy man, sustained a simple fracture of the right thigh just below the middle, the consequence of a fall from a scaffold. He also had a flesh wound of the chin which was sewed up, and dressed antiseptically. He was taken at once to the clinique, where the limb was dressed and ice applied. The patient complained very little of pain, although quite conscious. This occurred Nov. 14, 1874. On the following day, Prof. C. in a clinical lecture on the case, mentioned that although free from fever, the previous day, his temperature that morning was nearly 103° F., and he gave a guarded prognosis. In the evening the temperature was almost a degree higher. Although at night he was free from pain, he was sleepless (?), and had at intervals of three hours doses of about one-eighth gr. each of morphia. Some hours after midnight he was found comatose, with deep and frequent respirations, accompanied by coarse râles. The chest was resonant on percussion. Pulse 100, full and strong. He was bled from the arm, but obtained no relief and died at half past seven, thirty-eight hours after the injury.

At the autopsy, both lungs contained air and fluid. The left side of the heart was contracted, and there were coagula in both sides. The vessels of the pia mater and of the brain were distended with blood, and in both of these were numerous small ecchymoses. There were extensive extravasations of blood at and about the seat of fracture, but the femoral vein and artery were not wounded. The microscope revealed throughout the lungs a firm injection of all the smaller arteries and the capillaries, with clear fluid fat. Likewise in the brain, corresponding to the small ecchymoses, were branching fatty emboli in the vessels. Fat was also in the vessels of the kidneys and liver. The fatty embolism of the lungs was considered a sufficient cause of death.

Prof. C. quotes the statement from Busch that fatty embolism begins in the first few hours after the injury. The difficulty of respiration would point to this condition. Possibly the slight sensitiveness to pain may also be symptomatic of it.

Shock cannot be held to explain a death after injury, when an interval of several hours of good condition has intervened between the injury and death. He concludes that fatty embolism is of essential significance only after injuries of the bones, and perhaps osteo-myelitis. We may, continues Prof. C., suspect this condition when after such injuries a rapid failure sets in, with interference with the circulation in the lungs ; and secondly, in the general capillary circulation, without other cause, and very shortly after the injury is received.

2. *Luxation of the Thumb Backward.* FARABEUF. (*L'Union Médicale*, No. 25.)

The original paper of this author is admirable, and the anatomical points involved are carefully discussed, many experiments having been made on the cadaver.

The essential cause of the irreducibility of luxations of the thumb backward, is the interposition of the glenoid ligament between the articular faces ; but the sesamoid apparatus to be found in this ligament and in especial the external sesamoid bone, are the chief factors.

The whole question is made to turn on the mode of the articulation of the sesamoid bones with the phalanx. The latter is so disposed in the articulation, that it will permit of extreme flexion, but not of extreme extension—cartilage cannot be applied to cartilage. The sesamoid bone and the phalanx (or rather the articular face of the latter,) form a right angle which F. compares to that of a table-leaf which can be lowered but cannot be raised to a higher level than that of the table itself.

If we suppose a movement of extension in the thumb sufficient to tear the glenoid ligament, where does the rent occur ? The mode of insertion of the glenoid ligament upon the metacarpus and the phalanx enables us to understand that the metacarpal insertion will first yield. In 100 experiments, F. has found the phalangeal insertion torn but once. It can then be accepted as a fact, that when the extension of the thumb is sufficient to rupture the glenoid ligament, the sesamoid bones will remain attached to the phalanx.

If, in the movement of extension, the sesamoid bone does *not* slide beyond the cartilaginous face of the metacarpus, the luxation is *incomplete*. If the phalanx is carried more or less further back upon the dorsum of the metacarpus, carrying with it the sesamoid bone, the luxation is *complete*. Finally, there is a third variety named *complex*, the mechanism of which is carefully studied, and which accounts for the irreducibility of certain luxations.

When the phalanx is dislocated upon the dorsum of the metacarpus, it is maintained in this position by the muscular button-hole formed by the sesamoid muscles. From this it results that if the thumb be bent downward, the sesamoid bone is reversed, so that its cartilaginous face looks upward, and it becomes situated behind the phalanx instead of in front of it. It is this inversion of the sesamoid bone which constitutes the *complex* luxation.

Given this disposition of the osseous surfaces, the difficulty lies in the impossibility of bringing the sesamoid bone down over the articular face. Reduction is possible in such cases only when the two articular surfaces are capable of separation to the extent of the length of the sesamoid bone—that is, to the extent of about six millimetres. But the intact portion of the ligaments resists this separation, so that the luxation becomes irreducible.

The reduction proposed by Farabeuf is based upon the reciprocal relation of the luxated surfaces. It is needful :

1. Never to flex the finger upon the metacarpus, for this may transform a *complete* into a *complex* luxation.

2. To commence by pressing the thumb backward if it has been depressed.

3. To seize the first phalanx with a pair of forceps, hold it at right angles to the metacarpus, and give it a movement in its totality from behind forward, so as to force off the sesamoid bone. It is the phalanx itself which does this.

4. If the luxation is several days old, to give it a lateral motion, so as to determine a rupture of the lateral ligaments.

5. In cases of irreducible luxation, to make subcutaneous section of the two lateral ligaments, and reduce afterward.

"En résumé," says the reporter of the Surgical Society, "M. Farabeuf seems to me to have demonstrated that the cause of irreducibility in thumb luxations, arises not only from the interposition of the glenoid ligament, but further and more particularly from the turning over of the external sesamoid bone, which becomes, as it were, buttressed upon the dorsum of the metacarpus. He has shown also the exceedingly important rôle of the ligaments and the muscular button-hole, though these are but secondary factors. He has produced before me upon the cadaver irreducible luxations ; and I have been in position to assure myself of the reality of the mechanism he describes. The author has collected everything available upon this subject from the literature of all nations, and has produced a work which displays erudition, critical comparison and much original observation. It is accompanied by a large number of figures ably executed by the author."

3. *Radical Cure of Hernia.* PROF. NUSSBAUM. (*Allg. Med. Central-Ztg.*, 18, 1876.)

The favorable results obtained by Lister's antiseptic treatment of lesions of the peritoneum encouraged the professor to attempt the cure of hernia by catgut sutures in a few cases where an operation was imperatively indicated by the impossibility of getting a truss to retain the rupture.

After a thorough evacuation of the bowels by cathartics, the patient was narcotized and his scrotal hernia was well exposed by a long incision through the integument and the other coverings. All adhesions of the hernial sac with the testicle and spermatic cord were severed, and after a careful reposition of the intestines contained in it, the sac was sewn up by catgut sutures as near the inguinal canal as possible.

One centimetre below these sutures the empty sac was cut off and the upper end of it, with the stitches, pushed into the abdominal cavity. The external wound was then closed by antiseptic silk sutures, and the case was treated by a strictly antiseptic dressing.

The healing process went on unattended by any untoward symptoms, and the immediate result was exceedingly satisfactory. Still the professor thinks it premature to venture a definite opinion on this operation.

4. *A Remarkable Case of Skin-grafting.* DR. AUG. REVERDIN. (*Deutsche Zeitschr. f. Chir.*, vi., 418.)

A woman, 21 years of age, while working in a factory was caught by her hair in the machinery and the whole scalp was torn off her head. This accident occurred in April, 1872, and on October 10, of the same year, she was admitted to Prof. Lücke's clinic, in Strassburg, with an immense granulating wound which measured 14 inches (35 centimetres) from the root of the nose to the back of the head, and 11 inches (28 centims.) from one ear to the other. The upper part of the left ear was torn off with the scalp. The surface of this wound exhibited pretty good granulations and a copious secretion of pus, and was surrounded by a stripe of cicatrized and very vascular skin, about one inch (2 to 2½ centims.) in width.

After eight days of dressings with strips of adhesive plaster, the granulations had a more satisfactory appearance and the grafting process could be commenced.

For excising the grafts, Dr. R. used a lance-shaped knife (like the iridectomy knife) the blade of which was concave at the upper and convex at the lower side. From the knife the excised grafts could easily be removed to the wound with a needle where they were secured in their places by plaster strips. When the grafts had taken, the surrounding granulations were touched by lunar caustic in order to favor the formation of epidermis. After seven months the whole wound was healed, and the head was covered with a sound cicatrix in which large blood vessels could be seen.

During the treatment the doctor made various experiments with transplanting pieces of the skin of dogs and rabbits, and also with larger pieces (one to two inches square) of human integument taken from the amputated leg of a boy. The latter transplantations were successful, while those made with animal skin all failed.

